# Effects of bedtime melatonin treatment on blood pressure and arterial stiffness: a three-month, randomized, placebo-controlled crossover study in individuals with type 2 diabetes mellitus

**DOI:** 10.1007/s00592-026-02750-0

**Published:** 2026-08-12

**Authors:** Esben Stistrup Lauritzen, Julie Støy, Niels Møller, Per Løgstrup Poulsen, Ulla Kampmann

**Affiliations:** 1 Steno Diabetes Center Aarhus, Aarhus University Hospital, Palle Juul-Jensens Boulevard 11, Indgang A, Aarhus N, 8200 Denmark; 2 Medical/Steno Research Laboratory, Department of Clinical Medicine, Aarhus University, Aarhus, Denmark; 3 Department of Endocrinology and Internal Medicine, Aarhus University Hospital, Aarhus, Denmark; 4 Department of Clinical Medicine, Aarhus University, Aarhus, Denmark

**Keywords:** Melatonin, Hypertension, Blood pressure, Diabetes, Circadian rhythm

## Abstract

**Aim:**

Blood pressure is regulated in part by circadian rhythms, and melatonin has been reported to exert blood pressure–lowering effects. The underlying mechanisms, however, remain unclear. We hypothesized that melatonin would lower blood pressure in individuals with type 2 diabetes with or without hypertension, and that this effect would be mediated by a reduction in arterial stiffness.

**Methods:**

We conducted a double-blinded, randomized, placebo-controlled crossover trial involving 17 individuals with type 2 diabetes. Participants completed two treatment phases: three months of 10 mg melatonin taken one hour before nighttime, and three months of placebo taken at the same time separated by a four-week washout period. On the last day of each period, participants underwent 24-hour ambulatory blood pressure monitoring, including measurement of arterial stiffness, using the Arteriograph 24. Fifteen participants completed the 24-hour ambulatory blood pressure monitoring (hypertension *n* = 6).

**Results:**

Melatonin treatment was associated with numerical, non-significant reductions in mean nighttime systolic blood pressure (-6 [-13:1] mmHg, *p* = 0.08, ≈ 5% decrease) and diastolic blood pressure (-5 [-11:1] %, *p* = 0.11). The pulse wave velocity as a measure for arterial stiffness was unaffected by melatonin treatment (*p* = 0.83).

**Conclusion:**

In this randomized placebo-controlled trial of individuals with type 2 diabetes, 3 months of treatment with 10 mg melatonin administered one hour before bedtime did not significantly affect blood pressure or pulse wave velocity. Although numerical reductions in nighttime blood pressure were observed, these hypothesis-generating findings were not statistically significant and warrant confirmation in larger studies specifically powered for blood pressure outcomes.

## Introduction

In healthy humans, blood pressure follows a diurnal rhythm with peak pressures during the morning hours and lowest pressures during sleep [1]. This rhythm is under influence of the suprachiasmatic nucleus, the primary clock in the human body, which communicates with peripheral targets such as the heart, vasculature, and kidneys through both autonomous nervous fibers and hormones [2–5]. The suprachiasmatic nucleus also connects to the pineal gland via noradrenergic fibers through the superior cervical ganglion, stimulating melatonin secretion during nighttime [6].

Exogeneous melatonin has garnered significant attention as a potential antihypertensive agent because its endogenous release aligns with the nighttime dip in blood pressure. The nighttime blood pressure dip does not occur in all individuals, known as “non-dippers”, and a previous meta-analysis found that the nighttime blood pressure was a superior independent predictor of cardiovascular events and total mortality compared to daytime blood pressure [7]. Previous research has indicated that melatonin increases the vagal tone on the heart and that it may have suppressive effects on the sympathetic tone [8]. Accordingly, melatonin before bedtime reduced nighttime systolic blood pressure in individuals with untreated essential hypertension [9]. Individuals with type 2 diabetes are at markedly increased risk of cardiovascular disease, with hypertension affecting approximately two-thirds of adults with diabetes in the United States [10]. Notably, non-dipping of nocturnal blood pressure occurs in about one-third of individuals with type 2 diabetes [11]. Effective blood pressure control is therefore of critical importance in individuals with type 2 diabetes. Although several studies have demonstrated blood pressure–lowering effects of melatonin [12–14], no trial has specifically assessed the impact of melatonin on blood pressure and arterial stiffness in individuals with type 2 diabetes. Arterial stiffness is a well-established, independent predictor of cardiovascular disease and can be reversed by interventions such as exercise, statins, and renin–angiotensin–aldosterone system (RAAS) blockade [15]. We therefore hypothesized that melatonin lowers blood pressure, at least in part, through a reduction in arterial stiffness

## **Methods**

*Design:* We conducted a randomized, double-blind, placebo-controlled crossover trial involving 17 individuals with type 2 diabetes mellitus, including seven with concomitant hypertension. Participants were treated with 10 mg of melatonin or placebo one hour before individual bedtime for 12 weeks followed by a washout period of four weeks. After washout, the participants received the study drug that they did not receive in the first treatment period and were treated for another 12 weeks.

*Inclusion criteria:* Clinical diagnosis of type 2 diabetes within the last 20 years, Caucasian race, male sex, BMI 25–35 kg/m^2^ at diagnosis and age 40–70 years.

*Exclusion criteria:* blood pressure > 160/100, > 3 daily antihypertensive drugs, > 3 daily antidiabetic drugs, insulin treatment, HbA1c > 65 mmol/mol, > 1 lipid lowering drug, albuminuria, shift work within the last year, serious chronic illness, travel across > 4 time zones planned within the next 6 months, diagnosis of a sleep disorder, use of melatonin on a regular basis, melatonin allergy, medically treated depression or anxiety disorders within the last 3 years, daily consumption of medicine that interacts with the pharmacokinetics of melatonin, daily consumption of selective serotonin reuptake inhibitors or tricyclic antidepressants.

*24-hour ambulatory blood pressure and arterial stiffness measurements:* During the last day of each 12-week treatment period, participants arrived for a full study day including detailed investigation of glucose metabolism, body composition and substrate metabolism [16]. At the end of each study day (at 3.00 pm), an ambulatory blood pressure measurement with an Arteriograph 24 (TensioMed, Budapest, Hungary) was initiated with estimation of blood pressure and arterial stiffness every 20 min for the next 24 h. The Arteriograph 24 (TensioMed, Budapest, Hungary) has been validated for blood pressure and pulse wave velocity measurements (a measure of arterial stiffness) previously [17, 18]. The inflation cuff was placed on the non-dominant upper arm of the participant and the participant was instructed to keep the arm still during measurements. Before the participants left the clinical research unit, at least one measurement was performed, to check that the cuff was placed correctly. Briefly, the Arteriograph measures the brachial blood pressure, then the cuff deflates and subsequently inflates again to 35 mmHg above the level of the previously measured systolic blood pressure where the pulse configuration is recorded [18]. The pulse configuration contains two systolic pulse peaks. The first peak is generated by the ejection of blood from the left ventricle. The second peak is generated by the reflection of the pressure wave from the aortic bifurcation. The return time (RT) can then be calculated as the time difference (ms) between the first and second pulse wave which is done automatically by the Arteriograph device [18]. The pulse wave velocity (m/ms) can then pragmatically be calculated as the ratio between the distance from the top of the aortic arch (chosen as the jugulum) to the aortic bifurcation (pragmatically chosen as the symphysis) divided by the return time. This distance was measured with a wooden caliper specifically developed for this purpose to avoid variations in the distance introduced by the body surface when measured with a tape measure. The pulse pressure (PP) was calculated as the difference between the systolic and diastolic blood pressure. The mean arterial pressure (MAP) was calculated as 1/3 x systolic blood pressure + 2/3 diastolic blood pressure. The aortic augmentation index (AAI) was calculated as 100 x the pressure difference of the pulse waves/PP [19]. Each participant was asked to fill out a simple sleep diary during each 24-hour measurement with registration of bedtime and wake-up time.

*Statistics*: All statistical analyses were performed in STATA 16.0 (StataCorp, Houston, TX, USA) and all the graphical work was performed in SigmaPlot 11.0 (Systat Software, San Jose, CA, USA). Data were analyzed with a linear mixed model using restricted maximum likelihood with Kenward-Roger degrees of freedom. The model included treatment (melatonin or placebo), time (nighttime or daytime), order of treatment (melatonin-placebo or placebo-melatonin order), visit (1st or 2nd visit) and the interaction between treatment and time as fixed effects. The participants and the interaction between participants and visit were included as random effects. Missing observations were not imputed; instead, all available observations contributed to the analysis under the assumption that data were missing at random. The validity of the model was checked by inspection of quantile-quantile plots of the standardized residuals and by inspection of scatter plots of the fitted vs. the predicted values. Log-transformations were performed when appropriate; back transformed data are presented as geometrical means [95% CI] and differences as percent difference [95% CI]. Nighttime and daytime were calculated from bed and wake-up times from the sleep diaries. Seven subjects failed to return the sleep diaries. Hence, these subjects were set to a common bedtime at 22:00 and wake-up time at 06:00 for both visits when calculating the mean nighttime blood pressure and pulse wave velocity (reported sleep times ranged from 22:00–23:00 and reported wake-up times ranged from 06:00–09:00 for the participants who did return the sleep diaries). Due to technical errors with the Arteriograph 24 device, ambulatory measurements of pulse wave velocity were totally missing from three participants. Generally, we had more individual missing data points for the pulse wave velocity estimations (≈ 55%) compared with blood pressure measurements (≈ 29%). Power calculations for the study were based on insulin-stimulated glucose uptake, which determined that 18 participants were needed [16].

*Ethics *All participants gave their written informed consent prior to enrollment in the trial. The study was conducted in accordance with the Helsinki declaration. The study was approved by the Danish Medicines Agency and by the local scientific ethics committee (EudraCT 2019-000213-3). The study was also monitored by the Good Clinical Practice Unit at Aarhus University and Aarhus University Hospital. As phase-1 trial protocols are not published on EudraCT, we also registered the trial at clinicaltrials.gov (NCT03859934).

## Results

Participants: To account for potential dropouts, we enrolled 23 individuals. Ultimately, 17 participants completed both treatment periods. One left the trial for personal reasons (during melatonin), one developed severe psychiatric disease (during placebo), one developed thrombocytopenia and elevated liver enzymes (during placebo), one had worsening of metformin-induced diarrhea (during melatonin), one was diagnosed with colorectal carcinoma (during placebo) and one was reclassified to autoimmune diabetes (during washout). Fifteen participants completed Arteriograph 24 measurements during both melatonin and placebo treatment and six of these had a diagnosis of hypertension prior to the trial, which was too few individuals for meaningful subgroup analyses. A summary of the characteristics of those participants that completed both measurements (*n* = 15) and relevant medication is presented in Table 1. No participants changed antidiabetic, antihypertensive or lipid lowering therapy during the 7 months of treatment/washout.

**Table 1:** Participant characteristics and relevant medication at baseline

Characteristics	
Age (years)	65 (54–67)
Hypertension (yes/no)	6/9
Office blood pressure (mmHg)	144 (126–146)/88 (75–94)
Height (m)	1.79 ± 0.07
Weight (kg)	93 ± 13
BMI (kg/m^2^)	29 ± 3.4
Creatinine (µM)	62 ± 12
Total cholesterol (mM)	3.8 (3.2–5.5)
HDL (mM)	1.2 ± 0.3
LDL (mM)	2.1 ± 0.6
Triglycerides (mM)	1.4 ± 0.7
HbA1c (mmol/mol)	48.3 ± 7.4
Antiglycemic drugs	
Metformin	14 (93)
SGLT2-inhibitors	2 (13)
DPP-IV inhibitors	4 (27)
GLP-1 agonists	1 (7)
Anticoagulants and antiplatelet drugs	
DOAC	1 (7)
Acetylsalicylic acid (75 mg)	1 (7)
Lipid lowering drugs (statins)	8 (53)
Antihypertensive drugs	
RAAS inhibitor	5 (33)
β-blocker	2 (13)
Thiazides	1 (7)
Calcium channel blocker	2 (13)

Participant characteristics (means ± SD or median (interquartile range)) and antidiabetic, anticoagulant, lipid lowering and antihypertensive treatment (number of participants receiving the drug (percentage of all participants)). *HbA1c *: Glycated hemoglobin. *BMI*: Body mass index, *DPP:* Dipeptidyl peptidase, *SGLT2:* Sodium-glucose co-transporter, *GLP:* Glucagon-like peptide, *RAAS:* Renin-angiotensin-aldosterone system, *DOAC:* Direct oral anticoagulants*. These data have previously been published *[16].

**Fig. 1:**
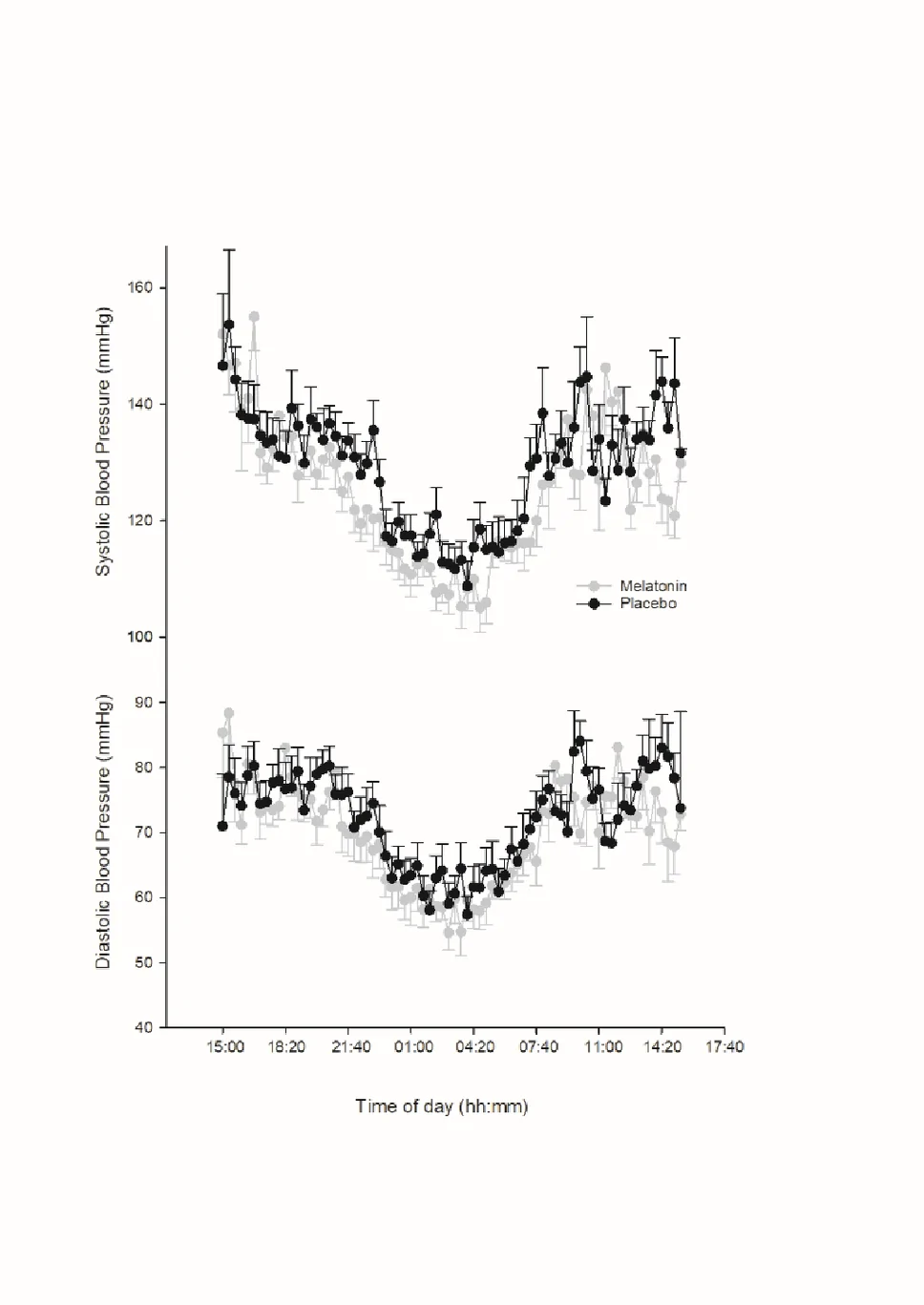
Systolic and diastolic blood pressure throughout the 24 ambulatory measurement period. Systolic and diastolic blood pressures (mmHg) during the 24-hour ambulatory blood pressure measurements. Grey curves = melatonin, black curves = placebo. Circles are means, error bars are standard errors

*Ambulatory blood pressure* (Fig. 1): Overall, melatonin treatment did not reduce systolic blood pressure (−4 [−10:2] mmHg, *p* = 0.18). When daytime and nighttime systolic blood pressures were analyzed separately, a significant physiological nocturnal dip was observed (−18 [−21:−15] mmHg, *p* = 0.00). Nighttime systolic blood pressure showed a non-significant numerical reduction with melatonin treatment (−6 [−13:1] mmHg, *p* = 0.08), whereas daytime systolic blood pressure remained unaffected (−2 [−9:5] mmHg, *p* = 0.50).

In a sensitivity analysis stratified by availability of sleep diaries, the numerical reduction in nighttime systolic blood pressure was − 3 [− 11:5] mmHg (*p* = 0.44) among the 8 participants who completed the sleep diaries and − 9 [− 21:3] mmHg (*p* = 0.12) among the 7 participants who did not return the sleep diaries.

For diastolic blood pressure, melatonin did not affect the overall diastolic blood pressure (−2 [−8:2] %, *p* = 0.37). When daytime and nighttime diastolic blood pressures were analyzed separately, there was a significant physiological nighttime dip in diastolic blood pressure (−18 [−21:−15] %, *p* = 0.00). Nighttime diastolic blood pressure showed a non-significant numerical reduction with melatonin treatment (−5 [−11:1] %, *p* = 0.11), whereas daytime diastolic blood pressure was unaffected (0 [−6:7] %, *p* = 0.99). For heart rate, mean arterial pressure (MAP), and pulse pressure (PP) please see Table 2.

**Table 2:** Daytime and nighttime blood pressure, heart rate, and vascular measures during placebo and melatonin treatment

	Placebo		Melatonin		Time x treatment	Treatment	Time	Daytime	Nighttime
	Daytime	Nighttime	Daytime	Nighttime					
Systolic blood pressure (mmHg)	134 [129–139]	117 [112–122]	132 [127–136]	111 [106–116]	0.21	0.18	0.00	0.50	0.08
Diastolic blood pressure (mmHg)	75 [70–79]	63 [59–67]	75 [70–79]	59 [56–64]	0.15	0.37	0.00	0.99	0.11
Heart rate (BPM)	77 [71–82]	66 [61–71]	78 [73–83]	69 [64–74]	0.55	0.25	0.00	0.66	0.20
MAP (mmHg)	120 [114–126]	103 [97–109]	119 [113–125]	98 [92–103]	0.18	0.33	0.00	0.81	0.14
Pulse pressure (mmHG)	58 [55 − 51]	53 [50–56]	56 [53–59]	51 [58 − 54]	0.80	0.16	0.00	0.30	0.18
Aortic pulse wave velocity (m/ms)	9.2 [8.7–9.6]	9.0 [8.5–9.5]	9.4 [9.0–9.9]	8.8 [8.3–9.3]	0.15	0.83	0.02	0.29	0.48
Aortic augmentation index (%)	20 [15–25]	22 [17–27]	20 [15–24]	20 [16–25]	0.63	0.72	0.32	0.95	0.58

Means [95% CI] for systolic blood pressure, diastolic blood pressure (back transformed), heart rate, mean arterial pressure (MAP), pulse pressure, aortic pulse wave velocity and aortic augmentation index stratified on treatment (melatonin or placebo) and time of day (daytime or nighttime). P values are provided for time x treatment interaction, treatment (melatonin vs. placebo for combined nighttime and daytime values), time (daytime vs. nighttime), nighttime (melatonin vs. placebo for nighttime) and daytime (melatonin vs. placebo for daytime)

*Arterial stiffness*: For pulse wave velocity there was a significant nocturnal decrease (−0.4 [−0.7:−0.1] m/ms, *p* = 0.02), but no effect of melatonin treatment (*p* = 0.83). For AAI please see Table 2.

**Fig. 2:**
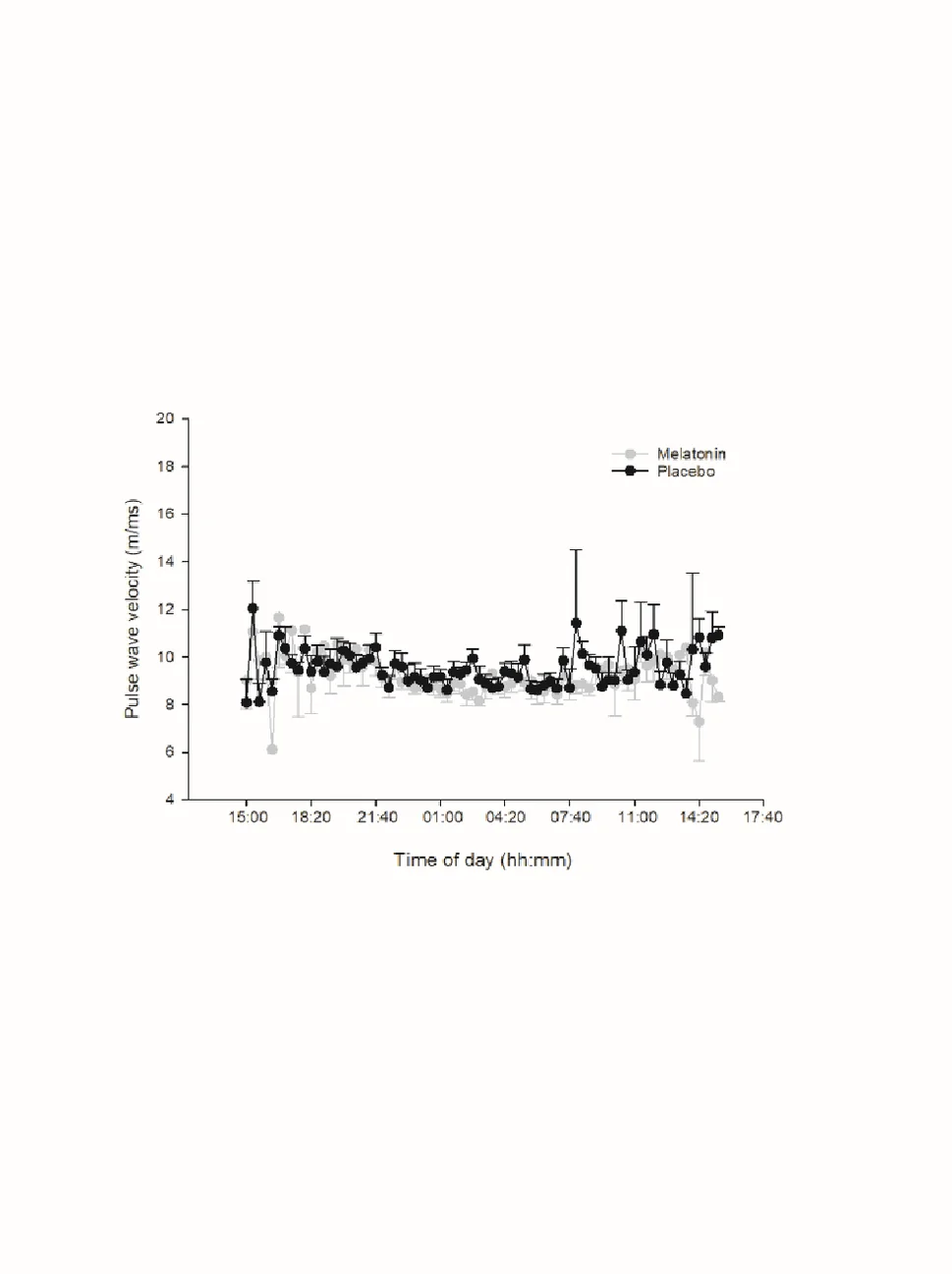
Pulse wave velocity throughout the 24 ambulatory measurement period. Pulse wave velocity (m/ms) during the 24-hour ambulatory blood pressure measurements. Grey curves = melatonin, black curves = placebo. Circles are means, error bars are standard errors

## Discussion

Overall, no significant effects of melatonin on ambulatory blood pressure or arterial stiffness were observed, irrespective of the time of day. However, our findings demonstrate that administration of 10 mg melatonin one hour before self-reported bedtime could contribute to lower nocturnal blood pressure in men with type 2 diabetes. Although the observed numerical reductions in nighttime systolic (6 mmHg) and diastolic (5% points) blood pressure did not reach statistical significance, the observed numerical reductions were of a magnitude that may be clinically relevant; however, given the limited sample size, these findings should only be considered hypothesis-generating. Notably, the numerical reduction was confined to nighttime measurements, coherent with an acute pharmacodynamic effect of melatonin, given its timing of administration in this study and the approximately one-hour half-life of exogenous melatonin [20]. As the study was not powered for blood pressure outcomes, the analyses are exploratory and the lack of statistical significance in nighttime blood pressures may therefore reflect a type II error due to the relatively low sample size. As such, the hypothesis that melatonin has beneficial effects on diurnal blood pressure rhythms, particularly in individuals at elevated cardiovascular risk who lack a nocturnal dip, cannot be rejected. These findings underscore the need for larger, adequately powered trials with blood pressure as the primary outcome.

Our findings regarding arterial stiffness should also be regarded as hypothesis-generating and interpreted with considerable caution due to the relatively small sample size and the high proportion of missing data (≈ 55% of all measurements) caused by technical difficulties with the Arteriograph 24 device, which substantially limits the precision of these analyses and underlines concerns about the clinical utility of the ArterioGraph 24 device [21]. Consistent with our findings, a study of women with insomnia receiving 2 mg prolonged-release melatonin for six weeks reported no effect on daytime arterial stiffness [22]. Similarly, a study in healthy postmenopausal women also found no impact of melatonin on arterial stiffness [23]. Neither of the studies demonstrated an effect on blood pressure, likely reflecting the inclusion of normotensive populations [22, 23]. Taken together, these findings demonstrate that if melatonin lowers blood pressure, it is unlikely to do so through reductions in arterial stiffness.

Thus, the mechanism underlying the previously observed blood pressure reduction [9, 12–14] remains unclear. Current and former night-shift workers have been shown to exhibit higher diastolic blood pressure compared with individuals performing daytime work, possibly reflecting desynchronization of internal circadian rhythms [24]. Interestingly, morning light therapy in night-shift workers has been shown to partially restore circadian alignment [25] and improve diurnal blood pressure control [26]. Taken together, the chronobiotic effects of the circadian hormone melatonin [27] may also contribute to beneficial blood pressure regulation; however, the molecular biological chronobiotic effects of melatonin was not investigated in the present trial.

Short sleep duration and insomnia are both associated with an increased risk of hypertension [28], and melatonin could theoretically improve blood pressure through improvements in sleep. However, in the present study melatonin had no measurable effect on either sleep quality or sleep duration [16].

Melatonin may also hold promise for patients treated with β-blockers, which are commonly prescribed for essential hypertension [29]. Because melatonin secretion is stimulated by noradrenergic innervation, β-blockers suppress nocturnal melatonin production and are frequently associated with insomnia [30]. In line with this, Scheer et al. demonstrated that hypertensive patients on β-blockers experienced improved sleep quality and quantity with melatonin compared with placebo [31].

Nevertheless, caution is warranted. Lusardi et al. investigated 47 individuals with essential hypertension in monotherapy with the calcium channel blocker nifedipine and found, that melatonin before bedtime actually increased systolic blood pressure with 6.5 mmHg and diastolic blood pressure with 4.9 mmHg throughout the 24-hour measurement period suggesting an unfortunate interaction between melatonin and calcium channel blockers [32].

The 10 mg dose was deliberately selected to achieve supraphysiological circulating melatonin concentrations during the nocturnal period, thereby maximizing the potential biological effects of melatonin treatment. Nevertheless, this dose exceeds those most commonly used in clinical practice [33], and it remains possible that lower doses, more closely mimicking physiological nocturnal melatonin secretion, may exert different cardiovascular effects.

Overall, additional randomized, double-blinded, placebo-controlled trials are warranted to clarify the role of melatonin on blood pressure. Future studies should specifically investigate the potential antihypertensive effect of melatonin, with particular attention to its impact on nocturnal hypertension in non-dippers, especially among individuals with coexisting diabetes and hypertension, a high-risk population with a high prevalence of non-dipping [11]. Future studies should include both male and female participants, be larger in scale and should be specifically powered to assess blood pressure outcomes and designed to evaluate both efficacy and safety.

The strengths of this study include its double-blinded, placebo-controlled design, the extended duration of melatonin administration, and the cross-over approach, which reduced inter-individual variability. Several limitations should also be acknowledged. First, a substantial number of missing values occurred, primarily in pulse wave velocity measurements. While the missingness was not believed to be related to treatment allocation or participant characteristics, some degree of systematic bias cannot be excluded. Larger studies using more robust methodology are required to clarify whether melatonin influences arterial stiffness. Second, the study population was restricted to Caucasian men with type 2 diabetes within a relatively narrow age and BMI range, limiting the external validity and generalizability of the findings to other subgroups. As sex differences in melatonin physiology may influence the cardiovascular effects, future studies should include women. Third, the study was not adequately powered to detect clinically meaningful effects of melatonin on blood pressure, as this outcome was secondary to the primary endpoint of insulin-stimulated glucose uptake [16]. Accordingly, the blood pressure analyses should be interpreted as exploratory and hypothesis-generating. Fourth, the inclusion of a combination of participants with or without preexisting hypertension may have masked blood pressure reducing effects of melatonin, as higher baseline blood pressure potentially results in larger reductions in blood pressure after initiation of many different antihypertensive agents [34]. Finally, the assignment of fixed sleep periods in participants without sleep diaries may have introduced some degree of misclassification in the definition of nighttime blood pressure. However, sensitivity analyses yielded results consistent with the overall findings, although these analyses were based on small sample sizes and should be interpreted with caution.

## Conclusion

Over a three-month period, melatonin treatment was associated with a numerical reduction in nocturnal systolic and diastolic blood pressure in individuals with type 2 diabetes, while arterial stiffness remained unchanged. Due to a low number of participants and lack of power, the study is exploratory and the findings are only hypothesis generating, and the results should as such be interpreted cautiously. Larger and adequately powered studies are required to determine whether melatonin offers true clinical benefit, particularly for individuals with non-dipping hypertension.

During the preparation of this work the authors used OpenAI ChatGPT 2025, GPT-5 in order to improve readability and language. After using this tool, the author reviewed and edited the content as needed and take full responsibility for the content of the published article.
